# The Establishment of a Treatment Protocol for the Reconstruction of Mid-Sized Defects in Lip Cancer Using Combinations with the Abbe Flap

**DOI:** 10.3390/jcm14072343

**Published:** 2025-03-28

**Authors:** Hyungjin Kweon, Hyunjoong Kim, Seoyeon Park, Euitae Choi, Yiqin Fang, Chunui Lee

**Affiliations:** Department of Oral and Maxillofacial Surgery, Wonju Severance Christian Hospital, Yonsei University, Wonju 26426, Republic of Korea; kwonhj0210@naver.com (H.K.); khj04537@naver.com (H.K.); psy960722@gmail.com (S.P.); chldmlxo96@gmail.com (E.C.); qin0302@naver.com (Y.F.)

**Keywords:** lip reconstruction, Abbe flap, perialar crescentic flap, staircase flap

## Abstract

**Background:** When defects occur in the lips due to conditions such as lip cancer, it is essential to reconstruct them both functionally and aesthetically, given the vital roles that lips play in communication, nutrition, and overall appearance. Various flap techniques are available for lip reconstruction, and the surgical method should be chosen based on the location and extent of the defect. **Methods:** This article discusses two cases of successful lip reconstruction using a combination of the Abbe flap and additional flaps, tailored to the extent of the defects. In case 1, a 52-year-old female diagnosed with angiosarcoma underwent a wide resection of 1/3 to 2/3 of her upper lip. Reconstruction was performed using an Abbe flap combined with a perialar crescentic flap. In case 2, a 54-year-old male with squamous cell carcinoma had more than 2/3 of his lower lip excised. Although the Bernard–Burrow–Webster flap is typically used for such cases, the patient was reconstructed with an Abbe flap combined with a staircase flap, addressing the limitations of traditional methods. **Results:** Both reconstruction surgeries achieved excellent aesthetic and functional outcomes. In case 1, the combination of the Abbe flap and perialar crescentic flap provided the patient with a natural appearance and preserved lip functionality. In case 2, the Abbe flap and staircase flap technique effectively restored lip function while avoiding complications such as microstomia and surgical scars associated with more conventional methods. **Conclusions:** The findings underscore that using the Abbe flap in combination with the perialar crescentic advancement flap or staircase flap can effectively restore both the functional and aesthetic integrity of the lips, particularly in cases involving significant tissue loss.

## 1. Introduction

The lips play a crucial role in communication, nutrition, and esthetics. Thus, when defects occur in the lip area due to congenital deformities, trauma, infection, cysts, benign tumors, or malignant tumors, it is necessary to reconstruct the lips functionally and esthetically. The best donor site for lip reconstruction is the lip tissue itself, which includes sensory and motor nerves, enabling the functional restoration of the orbicularis oris muscle. For the lower lip, maintaining the continuity of the orbicularis oris muscle is essential for its function [1].

The standard treatment for lower lip squamous cell carcinoma is surgical resection [2]. Typically, normal margins of more than 1 cm are included, resulting in larger defects [3]. For lesions smaller than 2 cm, resection in a “V” or “W” shape is performed, but for larger lesions, various flap techniques should be used based on the lesion’s location [4]. However, the treatment of defects that comprise more than two-thirds of the lip is challenging [5].

Various techniques for lip defect reconstruction have been discussed previously, including the Karapandzic flap (1974) [6], Abbe flap (1898) [7], Johanson staircase technique (1974) [8], Estlander flap (1872) [9], Gillies–McGregor flap [10,11], Bernard–Burrow–Webster flap (1960) [12,13], and fan flap (1920) [14]. Each method has its advantages and disadvantages, making it important to select the appropriate technique based on the defect’s location and extent.

This article introduces the use of the Abbe flap combined with the perialar crescentic advancement flap or staircase flap to effectively and functionally reconstruct lip defects in two cases.

## 2. Materials and Methods

### 2.1. Case 1

**Figure 1:**
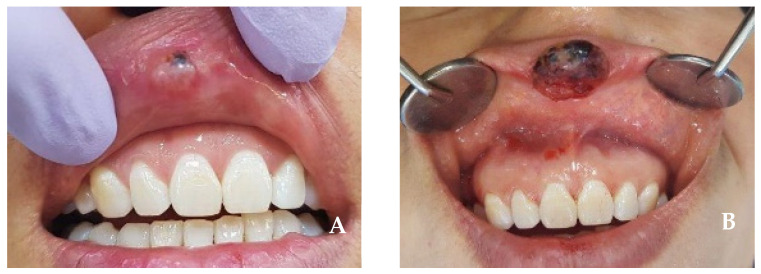
(**A**,**B**) Intraoral view at first visit. The tumor encompasses nearly 1/3 of the upper lip, located on the midline.

**Figure 2:**
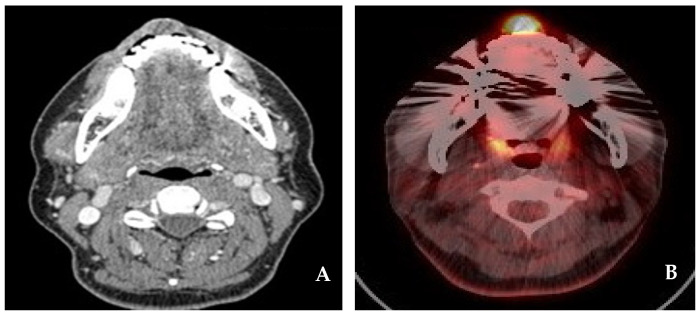
(**A**) CT neck(e). A well-defined hypodense mass with a size of 1.5 cm × 1.0 cm on the upper lip. (**B**) PET-CT. A focal metabolic lesion on the upper lip.

A 52-year-old female patient presenting with an enlarging tumor-like lesion in the central upper lip visited our oral and maxillofacial surgery department. An incisional biopsy under local anesthesia confirmed angiosarcoma. Clinical and imaging studies, such as CT and MR, revealed a tumor-like lesion of approximately 1.5 cm × 1.0 cm in the central upper lip, excluding the commissure (Figure 1 and Figure 2). No bony invasion of the adjacent maxilla or significant lymph node involvement were noted, and there were no distant metastases shown on PET-CT. The diagnosis was cT1N0M0Gx, stage I angiosarcoma, and surgical treatment was planned.

Surgical treatment was performed under general anesthesia by a single surgeon at Wonju Severance Christian Hospital in January 2018. A wide resection of the primary lesion in the upper lip was carried out. To reconstruct the defect, a combination of the Abbe flap, based on the inferior labial artery, and the perialar crescentic flap was used.

**Figure 3:**
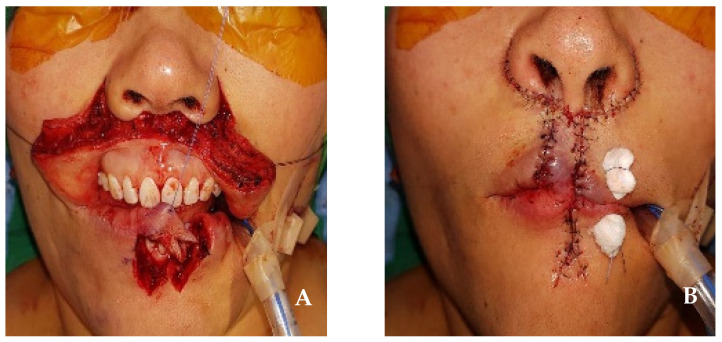
(**A**) The incision of the Abbe flap and perialar crescentic flap. The Abbe flap was harvested from the contralateral side of the lower lip. (**B**) After the closure of the skin incision.

**Figure 4:**
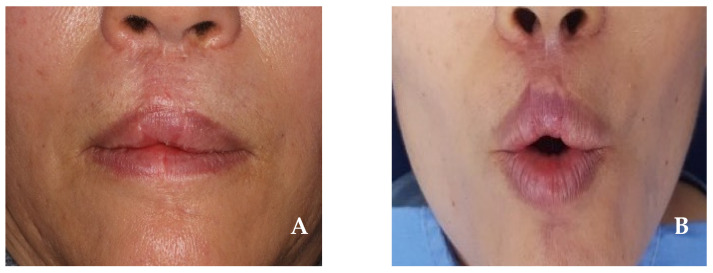
(**A**,**B**) Six months after surgery. Neither microstomia nor significant surgical scars appeared, and the function of the orbicularis oris muscle was well preserved.

In the first step, the resection of the upper lip was performed to approximately 2.5 cm × 1.5 cm, including the safety margin; this was equivalent to one-third to two-thirds of the total lip size. At this time, the tissue was incised in a crescent shape around both nasal wings to facilitate the advancement of the surrounding lip tissue during postoperative reconstruction. In the second step, the Abbe flap was harvested from the lower lip. A flap measuring 1.5 cm in horizontal length and 1.5 cm in vertical length was harvested from the contralateral side of the lower lip while preserving the inferior labial artery. At this time, in order to reconstruct the philtral crest of the upper lip and connect the Abbe flap to the columella during reconstruction, a “W”-shaped flap was harvested from the midsection of the lower lip, and the flap was rotated to the central upper lip defect (Figure 3A). Primary suturing was then performed using a layered suture. In the third step, the perialar crescentic flap was advanced to the Abbe flap, and a layered suture was performed (Figure 3B). As a final step, 26 days after surgery, the inferior labial artery connecting the Abbe flap pedicle was cut, separated, and sutured. At the 6-month follow-up, no recurrence of angiosarcoma in the primary site or lymph node was observed. The upper lip reconstruction showed no microstomia or significant surgical scars, and the esthetic appearance of the philtrum was well preserved. Functionally, the continuity of the orbicularis oris muscle was maintained, and normal muscle function was observed (Figure 4A,B).

### 2.2. Case 2

**Figure 5:**
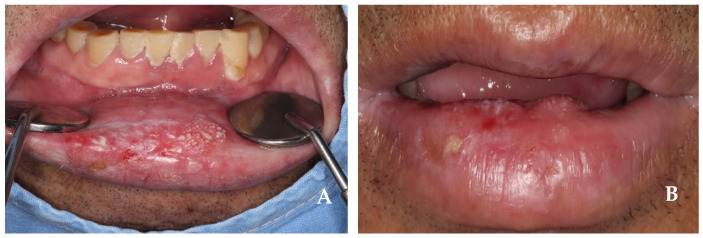
(**A**,**B**) Intraoral view at first visit. The tumor encompasses nearly 2/3 of the lower lip, located on the midline.

**Figure 6:**
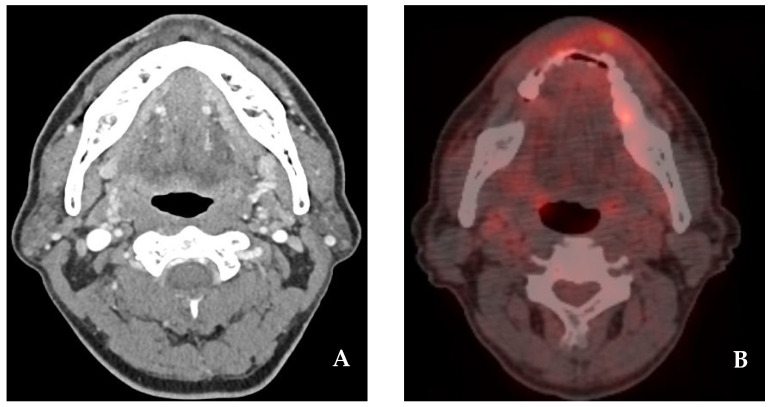
(**A**) CT neck(e). The image enhances the mass lesion with a size of 3.0 cm × 1.0 cm on the lower lip. (**B**) PET-CT. Focal metabolic lesion on the lower lip.

A 54-year-old male patient with an ulcerative lesion in the central lower lip that had developed in the past two months visited our oral and maxillofacial surgery department. An incisional biopsy under local anesthesia confirmed squamous cell carcinoma. Based on clinical and imaging studies, such as CT and MR, a tumor-like lesion measuring approximately 3.0 cm × 1.0 cm was confirmed in the central lower lip, excluding the commissure (Figure 5 and Figure 6). There was no bony invasion of the mandible or significant lymph node involvement, and no distant metastases were observed on PET-CT. The diagnosis was cT2N0M0, stage II squamous cell carcinoma, and surgical treatment was planned.

Surgical treatment was performed under general anesthesia by a single surgeon at Wonju Severance Christian Hospital in September 2023. A wide resection of the primary lesion in the lower lip and a level I–III selective neck dissection were carried out. Then, in order to reconstruct the defect, an Abbe flap, based on the superior labial artery of the upper lip, and a staircase flap, which forms a stepped flap on the side of the lower lip, were applied together.

**Figure 7:**
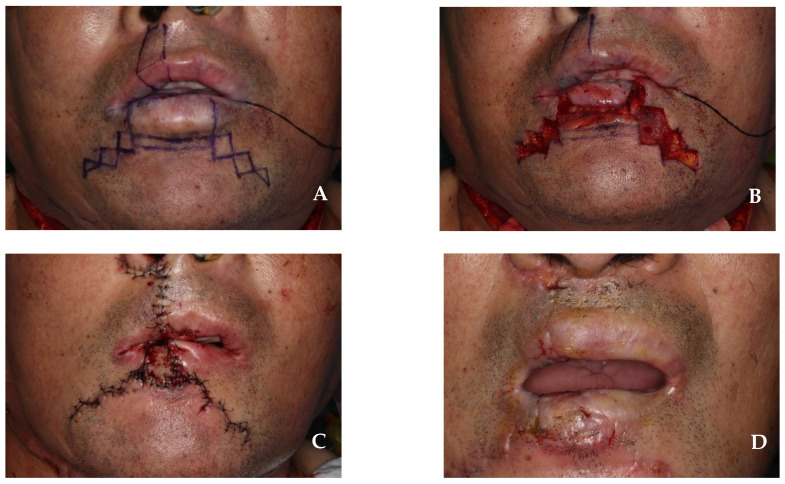
(**A**) Design combining Abbe flap and staircase flap. The Abbe flap was designed to be harvested from the contralateral side of the upper lip. (**B**) The incision of a staircase flap at the subcutaneous level. Orbicularis oris muscle is preserved. (**C**) After the closure of the skin incision. (**D**) After the division of the Abbe flap pedicle, 16 days after surgery.

**Figure 8:**
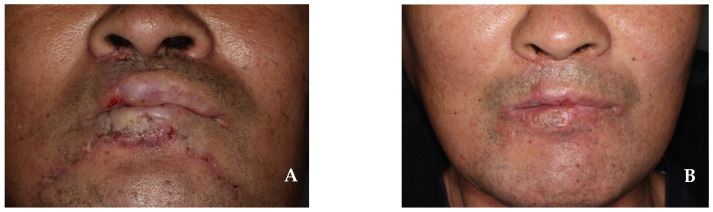
(**A**) At 1.5 months after surgery. (**B**) At 4 months after surgery. Neither microstomia nor significant surgical scars appeared.

The resection of the lower lip was performed to approximately 4.0 cm × 1.5 cm, including the safety margin; this corresponded to more than two-thirds of the total lip size. The first step involved creating a staircase flap with step widths of 0.3 cm, 0.6 cm, 0.9 cm, and 1.2 cm, while preserving the orbicularis oris muscle (Figure 7A,B). In the second step, an Abbe flap measuring 1.5 cm × 1.5 cm was harvested from the contralateral side of the upper lip, avoiding the philtrum and preserving the superior labial artery. The flap harvested from the upper lip was rotated to the center of the lower lip defect, and primary suturing was performed using a layered suture. In the third step, the staircase flap was advanced over the Abbe flap and a layered suture was performed (Figure 7C). As a final step, 16 days after surgery, the superior labial artery connecting the Abbe flap pedicle was cut, separated, and sutured (Figure 7D). The 6-month follow-up showed no recurrence of squamous cell carcinoma in the primary site or lymph node. The lower lip reconstruction demonstrated normal sensation, speech function, and muscle function, with no microstomia or significant surgical scars (Figure 8).

## 3. Results and Discussion

The reconstruction of the lips requires the consideration of both the functional and esthetic aspects of the lips. The lips play a vital role in maintaining the esthetic balance of the face. As one of the key facial features, the shape and contour of the lips have a substantial impact on the overall appearance of the face. Thus, lip reconstruction is crucial to the preservation or restoration of a natural and balanced appearance. Additionally, the lips are used in functional activities, such as speech and swallowing. They aid in articulation during speech and support the process of swallowing food. Consequently, the functional recovery of the lips is vital for ensuring clear speech and a proper nutritional intake. Lip reconstruction goes beyond merely improving appearance; it has a significant impact on the patient’s quality of life and enables the restoration of daily functions [6].

**Figure 9:**
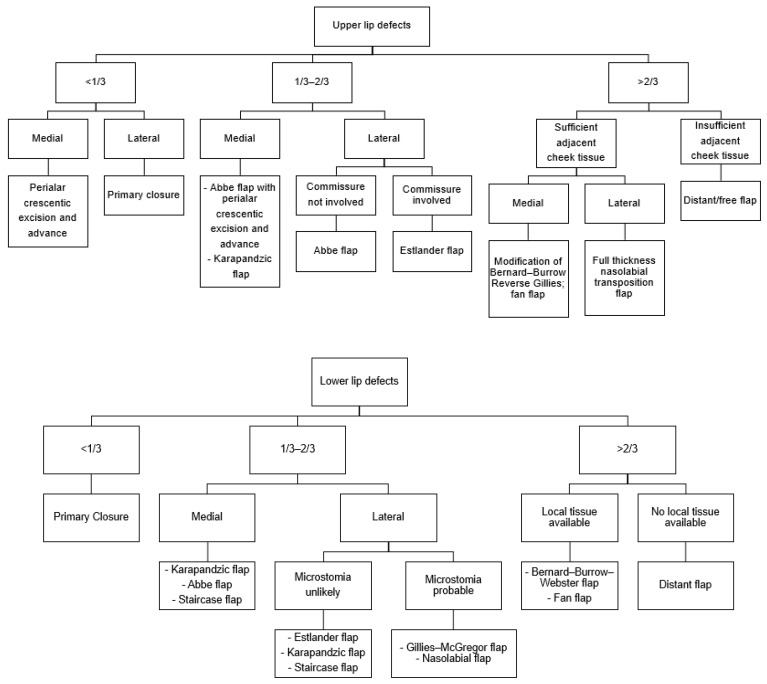
Treatment flow chart for lip reconstruction based on the size and location of the defect [1].

When selecting a surgical method for the successful reconstruction of lip defects, the size and location of the defect are primary considerations (Figure 9). For small defects that comprise less than one-third of the lip, direct closure with “V”- or “W”-shaped excisions without flap usage is possible. In contrast, defects involving one-third to two-thirds of the lip require the use of surrounding tissues for reconstruction [1]. Techniques such as the Karapandzic flap (1974) [6], Abbe flap (1898) [7], Johanson staircase technique (1974) [8], Estlander flap (1872) [9], and Gillies–McGregor flap [10,11] fall into this category. The Bernard–Burrow–Webster flap (1960) is used for defects that involve more than two-thirds of the lip, but it may result in unsatisfactory sensory and functional outcomes, potentially leading to issues such drooling. Severe scarring, microstomia, and restricted mouth opening are also common complications [12,13]. The fan flap (1920) [14] is also used to reconstruct large lip defects, offering tissue that closely resembles the original in quality and texture. However, the rotation of these flaps can lead to microstomia and alter the anatomy of the commissures, sometimes necessitating commissurotomy for correction. Moreover, this technique may distort the orbicularis oris muscle fibers, which can result in an incomplete functional recovery and the loss of sensation [15].

**Figure 10:**
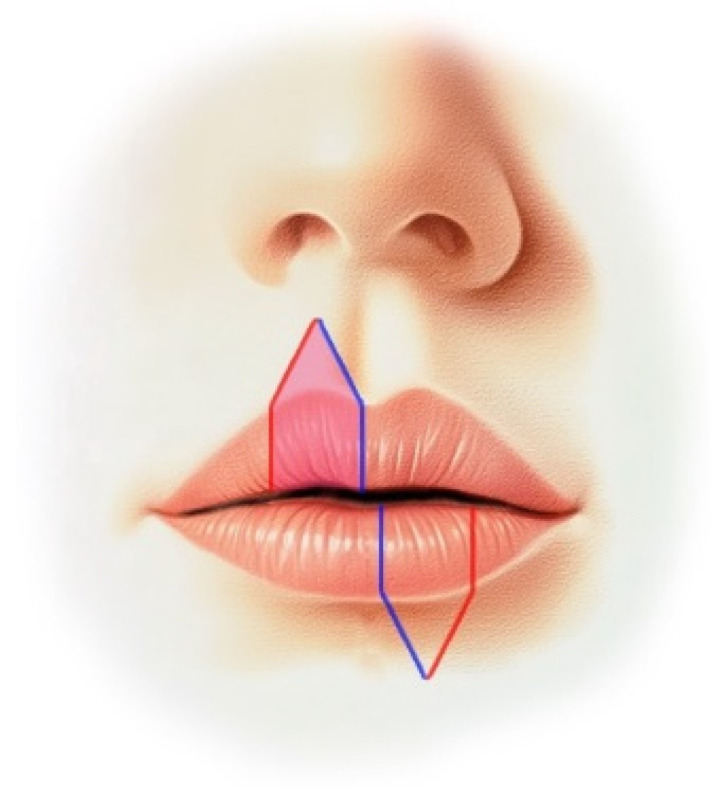
A flap is harvested from the contralateral side of the upper lip and rotated so that the red line aligns with the red line, while the blue line aligns with the blue line, in order to reconstruct the defect area of the lower lip.

The Abbe flap, designed by Robert Abbe in 1898, is a full-thickness flap used for the reconstruction of defects that involve one-third to two-thirds of the upper or lower lip. It is used when the size of the defect is one-third to two-thirds of the total lip size, and it includes the labial artery, branching from the facial artery. This flap is designed to minimize the mismatch in length between the lips, with a width that is half the defect’s width and a height that is equal to the defect’s height. Harvesting a flap from the other lip on the opposite side of the defect and rotating it for reconstruction is an additional method specifically designed to minimize lip asymmetry (Figure 10). In this approach, the portion of the flap that is longer is intentionally matched and directed toward the longer area of the lips, while the shorter segment of the flap is aligned with the shorter area. This tailored method ensures that the lengths of the lips are balanced effectively, minimizing any existing asymmetry. The Abbe flap is advantageous as it reconstructs the entire thickness of the lip tissue, enabling the restoration of the orbicularis oris muscle [16]. Furthermore, the redistribution of nerve tissue occurs in the muscle layer of the Abbe flap, so there is a lower reduction in sensation [17]; in addition, the donor site can be closed primarily, which reduces scarring. However, a secondary surgery for pedicle separation is required after 14 to 28 days [18].

Various methods can be used to treat full-thickness defects that comprise less than one-third of the upper lip, with the most popular being the ipsilateral nasolabial flap. However, this flap can result in dog ears and may appear less natural in men due to hairless skin being moved to a hairy area. The perialar crescentic flap, designed by Webster in 1955, involves the excision of a crescent-shaped area of skin around the ala and the advancement of a flap from the cheek or lateral part of the lip to reconstruct the upper lip defect. This method is esthetically pleasing as the scar is placed around the ala, and unlike the ipsilateral nasolabial flap, it avoids unnatural results by matching similar tissues [19]. The staircase flap, designed by Johanson in 1974, involves the advancement of a flap from the side of the lip and chin in a stepwise manner to reconstruct the defect [5]. This technique is used for defects that comprise one-third to two-thirds of the lip, and offers the following advantages: it is able to hide scarring in the mentolabial fold, maintains the commissure’s shape, and does not affect the surrounding muscles, such as the depressor labii inferioris or orbicularis oris muscle [1].

In this case study, the reconstruction of lip defects was performed using a combination of the Abbe flap and various techniques. In the first case, one-third to two-thirds of the upper lip, including the safety margin, was resected. This originally corresponds to the indication for the Karapandzic flap; however, in this case, microstomia may have occurred [20]. In addition to this, major complications include severe scarring, sensory abnormalities, the disharmony of the upper and lower lip, and the rounding of the corner of the mouth [21]. Accordingly, we performed reconstruction using a combination of the Abbe flap and perialar crescentic advancement flap. This approach enabled the above complications, such as microstomia and severe surgical scarring, to be avoided; meanwhile, the esthetic appearance of the philtrum area was maintained. No significant sensory or functional impairments were observed.

In the second case, more than two-thirds of the lower lip, including the safety margin, was resected. This is the original indication for the Bernard–Burrow–Webster flap or fan flap, but in this case, the various complications mentioned above may have occurred. Accordingly, various other methods for the reconstruction of large lip defects have been discussed, but the results are usually unsatisfactory; therefore, there is currently no standard treatment [4,22]. For example, when the Karapandzic flap and the Bernard–Burrow–Webster flap were used together for patients with defects comprising more than two-thirds of the lip, there was no decrease in the sensation and function of the lower lip, which is an existing complication of the Bernard–Burrow–Webster flap; however, microstomia could not be completely avoided [23]. Using a free flap, rather than a local flap, was also considered. Free flap reconstruction, such as the forearm flap, has demonstrated remarkable outcomes due to its dependable blood supply, adequate tissue volume, and its ability to restore lip motor and sensory functions by incorporating vascularized nerves and muscles [24]. However, it ultimately demonstrates limitations in the reproduction of oral function and a maximal aesthetic outcome because it differs from oral tissue [25,26].

In this case, the reconstruction of the lower lip was performed using a combination of the Abbe flap and the staircase flap. Although this method has not been widely studied, it effectively avoided complications, such as microstomia and severe scarring, while preserving sensory and functional outcomes. The scars formed in areas that could be concealed by facial hair in men, making it a more esthetically pleasing option.

In summary, the treatment protocol for the reconstruction of middle-sized defects in the lip using a combination of the Abbe flap is as follows:

1. Abbe flap design: the flap should be designed to minimize the length mismatch between the lips, with a width of half the defect’s width and a height equal to the defect’s height.
2. Harvesting technique: harvesting a flap from the other lip on the opposite side of the defect is recommended to effectively reduce lip asymmetry.
3. Flap preservation: the Abbe flap must be harvested with the full thickness of the lip tissue, ensuring the preservation of the labial artery, which branches from the facial artery.
4. The combination of flaps: combining the Abbe flap with a staircase flap may be particularly advantageous for patients with defects comprising more than two-thirds of the lower lip, excluding the commissure.
5. Revision surgery: revision surgery may be necessary for minor corrections to achieve maximum aesthetic results, typically performed 3–6 months after initial surgery.

In conclusion, the reconstruction of the lips necessitates the careful consideration of both functional and aesthetic aspects. It is essential to achieve a balanced reconstruction that addresses both the functionality of the lips and their appearance, ensuring a harmonious lip structure. This highlights the importance of selecting the appropriate technique based on the defect’s size and location. Using the Abbe flap and staircase flap in combination could be a useful option for patients with a defect comprising more than two-thirds of the lower lip, excluding the commissure, and caused by, for example, malignancy.

## Data Availability

All the research data are available upon request from the corresponding author.
